# On the Acetate of Lead in Alvine Discharges

**Published:** 1846-04

**Authors:** 


### SELECTIONS

FROM

### AMERICAN AND FOREIGN JOURNALS.

---

*On the Acetate of Lead in Alvine Discharges.*—Dr. Corrigan thus expresses himself in reference to the powers of this medicine in diarrhœas occurring towards the close of fever:

"Since I received a communication from Dr. Bardsley, recommending the trial of acetate of lead in that form of diarrhœa which comes on towards the termination of fever, and generally ends in ulceration of the Peyerian glands, I have made several clinical experiments with the view of ascertaining the powers of this remedy. I have found it exceedingly useful in controlling superabundant secretion in numerous instances. I have experienced the best effects from its employment, not only in the diarrhœa which accompanies ulceration of the mucous glands, but also in that species of diarrhœa which occurs at an earlier period of fever, and by means of which nature attempts the relief of intestinal congestion. Of its great value in the treatment of cholera I have already spoken; indeed, I do not know of any remedy by which inordinate fluxes from the bowels, whatever may be their nature, are so efficiently treated. The same remarks will apply to super-secretion from the lungs. In cases of phthisis attended with such copious secretion as to threaten suffocation, it is very beneficial: its effects are equally remarkable in chronic bronchitis with copious expectoration, and you are all aware of the great efficacy it possesses in checking hæmoptysis. I have been in the habit of prescribing it in combination with laudanum and wine vinegar; the latter I have added on the recommendation of Dr. Thompson, of London."—*Medical Times.—Ranking's Abstract.*

